# Using Advanced Tri‐Axial Accelerometer Data to Improve Behavioral Time Budgets and Bioenergetic Estimates of Wintering Lesser Scaup

**DOI:** 10.1002/ece3.72868

**Published:** 2026-01-05

**Authors:** Hannah L. Schley, Christopher K. Williams, Josh Homyack, Bill Harvey, Glenn H. Olsen, Sharon Johnson

**Affiliations:** ^1^ Department of Entomology and Wildlife Ecology University of Delaware Newark Delaware USA; ^2^ Maryland Department of Natural Resources Cambridge Maryland USA; ^3^ Retired Maryland Department of Natural Resources Cambridge Maryland USA; ^4^ Eastern Ecological Science Center, U.S. Geological Survey Laurel Maryland USA

**Keywords:** *Aythya affinis*, daily energy expenditure, lesser scaup, machine learning, scanning observations, time‐activity budgets, tri‐axial accelerometer data

## Abstract

Wildlife behavior studies have provided vital information towards understanding the natural histories of wildlife species and identified crucial components regarding their habitat and metabolic needs. For many species, typical behavioral data are collected using diurnal scans that have limitations in both when and where the data can be collected, ultimately leading to biases in behavioral patterns. With technological and analytical advancements of radiotechnology, behavior data can be collected more often and over larger spatial scales than with traditional methods. This study compares the behavioral time budget estimates between two different observational methods: ground‐truthed diurnal scanning observations and 24‐h tri‐axial accelerometer (ACC) GPS/GSM transmitter data that were classified using machine learning. We used the time budgets produced from the two methodologies and calculated the daily energy expenditure (DEE) for wintering Lesser Scaup ( *Aythya affinis* ) to explore the implications of biased behavioral data. We found significantly more feeding and less flight behavior of birds in the ACC data than in the visual scanning data. Using the ACC behavior proportions of the two most energetically demanding behaviors (feeding and flying), we found that feeding occurred 42% more during the day and flying occurred 23% more during the night. Lastly, we identified that the DEE estimated using the diurnal scanning observations produced a significantly lower estimate than with the 24‐h ACC data. This advanced way of interpreting wildlife behavior patterns can increase our understanding of wildlife species' natural history and make improved decisions regarding wildlife conservation and management. Incorporating this new technique of wildlife behavioral observations, we provided a new framework to expand our current knowledge of wintering waterfowl behaviors and energetic needs that can be adapted to research the vast intricacies of wildlife behavior.

## Graphical abstract

We compared two observational methods, traditional ground truthed diurnal scanning observations and 24‐h tri‐axial accelerometer GPS/GSM transmitter data, for calculating behavioral time budgets and estimated the daily energy expenditure of wintering Lesser Scaup. While our primary aim of this research was to improve our understanding of wintering behavioral patterns and to apply progressive decision tree analysis when deciphering behavioral observations, it also poses as an example of one of the many potential questions that can be answered when using GPS/GSM transmitters, often used for wildlife movement studies, and to act as a tool for other researchers that are studying high resolution behavioral data. Additionally, this research expands our work by incorporating new technology, techniques, and analysis to calculating behavioral time budgets and daily energetic estimates while simultaneously addressing the incomplete bias of past research and literature regarding this topic.

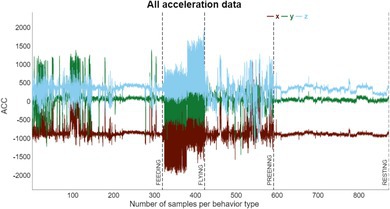

## 1 Introduction

Investigating behavioral patterns is invaluable to understanding wildlife species' natural history, conservation and population needs (Martin 1998). An understanding of behavioral patterns permits researchers to quantify wildlife daily activities including daily movement (Martin 1998), habitat use and resource selection (Pedersen et al. 2011), responses to environmental stimuli and environmental changes (Bro‐Jørgensen et al. 2019), impact of human disturbance (Owen et al. 2017), and their subsequent impacts on daily energetic costs (Wilmers et al. 2017). The energetic demand by wildlife species varies seasonally (Kautz et al. 1982) and can have substantial cross‐seasonal effects on reproduction and population levels (Sedinger and Alisauskas 2014). Therefore, to meet peak body condition for reproduction, it is imperative that wintering habitats contain ample food resources for individuals as this is a fundamental determinant of fitness and overall population stability (Ankney and MacInnes 1978; Afton and Ankney 1991; Tinkler et al. 2009).

The winter food limitation hypothesis states that the availability of food for energy and nutrients is the primary factor limiting waterfowl populations during winter and migration (Krapu and Swanson 1975; Sherry et al. 2005). To maintain and increase available wintering food resources for the conservation of waterfowl populations, biologists must consider the amount of energy present in the landscape. This is often modeled by using the energy supply and demand in the form of daily energy expenditure (DEE) of waterfowl species to estimate the carrying capacities of the wintering grounds (Livolsi et al. 2015). DEE represents the required energy needed to support an individual bird for 1 day and is calculated by (1) summing the basal metabolic rate (BMR), (2) thermoregulatory requirements at a specific lowest critical temperature, and (3) the energetic cost of daily behaviors such as flying, feeding, and swimming (McKinney and McWilliams 2005), also known as time‐activity budgets (Paulus 1988; Crook et al. 2009).

Instantaneous scan sampling is a data‐collecting technique which allows an observer to record current behavior patterns displayed by individuals at preselected moments in time and is typically used to record behaviors of individuals through direct observation as well as activities of groups, individual behavior synchronization and flock size, and spatial relationships in populations (Engel 1996). This technique can be advantageous because it is less obtrusive, inexpensive, and can produce large amounts of data over a short period of time (Paolisso and Hames 2010). Although scanning methods have often been used to estimate behavior patterns and DEE (Bergan et al. 1989; Ladin et al. 2011; Heise et al. 2019), there are limitations to this methodology that can bias behavior proportions and result in inaccurate DEE estimates. First, if behaviors cannot be continuously observed using scanning methods over a 24‐h period, at night for example, time budgets may not be reflective of the full period by missing important observations such as nocturnal behaviors (Wang et al. 2015). Because waterfowl movement and foraging behavior differs between diurnal and nocturnal time periods (Ydenberg et al. 1984; Jones et al. 2014; Heise et al. 2019), observing daily behaviors on a 24‐h cycle is vital for calculating energy estimates. Second, if a wildlife species occupies habitats where conducting scan samples is logistically difficult or impossible (e.g., open water or dense vegetation) inaccurate or incomplete behavior estimates can occur. Other methods, such as live‐stream web cameras, have been used to overcome these limitations; however, in areas such as the Chesapeake Bay, these methods still pose obstacles since collecting the data would require deploying cameras in a tidal environment that can cause unrepairable damage or lost equipment (Wood et al. 2025).

To counter the potential visual and spatial bias associated with scan samples, the use of *x*‐*y*‐*z* tri‐axial accelerometer (ACC) data provided by Global Positioning System‐Global System for Mobile Communications (GPS/GSM) transmitters can potentially help determine wildlife behavior. This technology has been instrumental in helping researchers and managers understand habitat and resource selection, spatial patterns, and home ranges for many wildlife species (Brown et al. 2012). Despite these advantages, ACC telemetry data are costly, reduces sample sizes, and may be difficult to statistically separate movement behaviors (Hebblewhite and Haydon 2010). Therefore, it is imperative to compare both field scanning observations and ACC technology when interpreting behaviors and calculating time budgets for DEE estimates.

Lesser Scaup ( *Aythya affinis* ) (hereafter scaup), populations in the United States have been declining steadily for over 20 years (Afton and Anderson 2001; Ross et al. 2012; US Fish and Wildlife Service 2024). While much focus has been devoted to their breeding‐ and reproductive ecology, there has been limited research conducted on limiting factors imposed by their wintering locations and behaviors (Austin et al. 2000). In winter, scaup rely on open bodies of water and forage on benthic organisms (i.e., bivalves such as clams and mollusks [*Bivalvia* spp.])to build up fat reserves and acquire the necessary energy for the following breeding season. The Chesapeake Bay and its tributaries comprise the largest estuary in the United States (Goetz et al. 2004) and offers ample open water habitat. However, much of the historically forested landscape has now been largely converted to urban/suburban residential communities, agriculture lands, and supporting infrastructure altering water quality and chemistry relative to historic parameters (Cooper 1995). Changes in water quality, along with changes in bottom topography, substrate composition, and submerged aquatic vegetation (SAV) species composition and availability may directly impact the abundance of the scaup's primary food resources in the benthic community and in turn impact wintering behaviors and habitat (Dauer et al. 2000; Najjar et al. 2010).

We calculated and analyzed wintering scaup behaviors using traditional diurnal observational scanning and 24‐h ACC technology. Our first goal was to compare the behavioral proportions during the diurnal and nocturnal portions of the day that were predicted using the ACC data, and we hypothesized that the high‐energy behaviors of feeding and flying would occur significantly more during the diurnal hours than in the nocturnal time period. We also hypothesized that the ACC method would observe feeding and flying behavior significantly more often than with the traditional scan sampling method. Second, we compared the diurnal and nocturnal energy expenditure calculated from the ACC behavior data and predicted that the nocturnal energy expenditure estimate would be significantly greater than the estimates from the diurnal time period. We also compared the difference between 24‐h ACC estimated DEE versus extrapolating diurnal behavioral scan sampling to the 24‐h period and the diurnal energy expenditure from the diurnal ACC data and the daylight behavioral scan samples. We hypothesized that the ACC DEE estimate would be greater than the scan sample DEE estimate for both the daylight comparison and 24‐h periods because the ACC would account for nocturnal movement behaviors as well as flight behaviors often not captured during observational scan sampling (Morton et al. 1989; Jeske and Percival 1995). Providing a full 24‐h behavior and energetic profile for scaup can be used to support future management and conservation decisions in their wintering areas.

## 2 Material and Methods

### 2.1 Study Area

**FIGURE 1:**
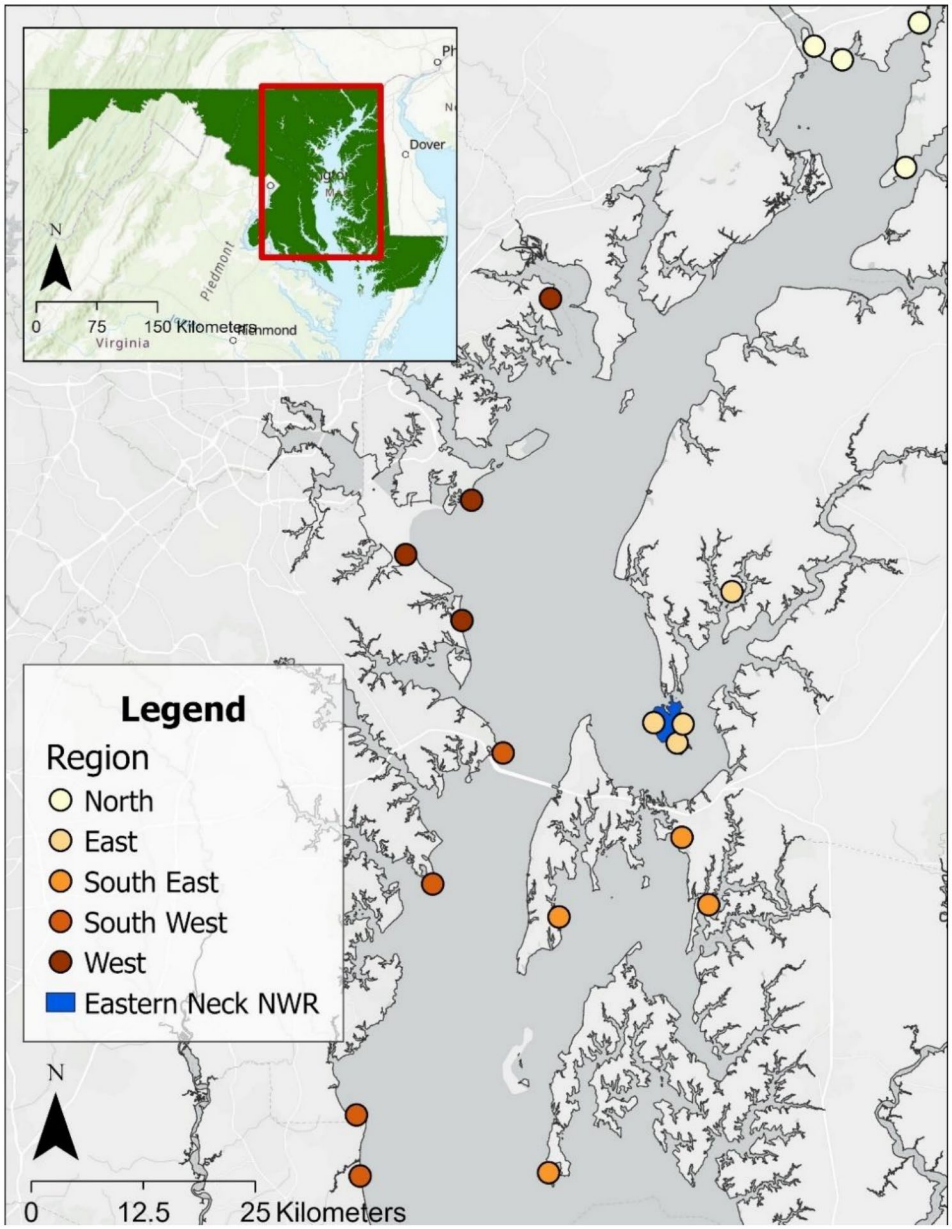
Focalized location (area outlined in red on inset map of Maryland, USA) showing five regions containing four observation locations distributed around the western and eastern shores of the Chesapeake Bay, Maryland, USA, used for behavioral scan sampling in January–March 2021 and 2023 and the December—March 2022 and 2023 trapping location at Eastern Neck National Wildlife Refuge, Rock Hall, Maryland, USA.

The Chesapeake Bay is the largest estuary in the United States and is located on the eastern coast surrounded by the states of Maryland and Virginia (Baird and Ulanowicz 1989; Reay and Moore 2009; Maryland State Archives 2021). For this study, we conducted our research in the Maryland portion of the Chesapeake Bay, USA (Figure 1) which encompasses approximately 11,603 sq. km with an average depth of 6.4 m and a maximum depth of 53.0 m (Reay and Moore 2009; Maryland State Archives 2021). There are two major salinity divisions that increase towards the south: oligohaline (0–6 g/kg) and mesohaline (6–18 g/kg; Baird and Ulanowicz 1989).

The Maryland portion of Chesapeake Bay region includes diverse land uses. The southeastern portion of Chesapeake Bay contains large expanses of saline wetlands comprised of smooth cordgrass ( *Spartina alterniflora* ), salt meadow grass ( *Spartina patens* ), big cordgrass ( *Spartina cynosuroides* ), needle rush ( *Juncus roemerianus* ), and marsh elder (*Iva fructescens*) (DeLuca et al. 2004). The northern region of the Chesapeake Bay is identified with freshwater wetlands consisting of narrow‐leaved cattails ( *Typha angustifolia* ), common reed ( *Phragmites australis* ), arrow arum ( *Peltandra virginica* ), rose mallow ( *Hibiscus moscheutos* ), oak‐tulip poplar ( *Liriodendron tulipifera* ) forests, and low‐density agriculture (DeLuca et al. 2004). In the central eastern shore region, commercial agriculture dominates the landscape (Keller et al. 1993; DeLuca et al. 2004; Szlávecz and Csuzdi 2007; Lamb et al. 2021) consisting of row crops, poultry production, and some pasture (DeLuca et al. 2004).

### 2.2 Behavioral Scan Observations

We conducted behavioral scan observations from late January–March in 2022 and 2023. These behavioral scans were conducted in five different regions that were located around the shore: southeast, east, north, west, and southwest (Figure 1). Each of these five regions were comprised of four observational points resulting in a total of 20 observational points (Figure 1).

Each week, we randomly selected four out of the five regions to conduct observational scans and randomly selected three of the four observational points from each specified region that were each scanned during two of four distinct time periods: morning crepuscular, morning—afternoon, afternoon—evening, and evening crepuscular. We determined the time periods using the package “suncalc v. 0.5.1” in RStudio v. 4.1.3 (McDuie et al. 2019; Thieurmel and Elmarhraoui 2022; R Core Team 2023) that uses astronomy, dates, and latitudes and longitudes of a specific area to calculate sunlight phases. Morning crepuscular was determined from dawn until initial sunrise, the morning—afternoon was calculated from initial sunrise to solar noon, afternoon—evening was calculated from solar noon until initial sunset, and evening crepuscular was from initial sunset to dusk. To make sure that each sampling region was represented for each time segment, every other day was sampled with the opposite time segment of the previous day. For example, on the first day of each week during the observations, we randomly selected one of the five sampling regions and then randomly selected three of the four observational points within that sampling region to visit during the morning crepuscular and afternoon segments. The following day, we randomly selected one of the remaining four sampling regions and its corresponding observational points to sample during the afternoon and evening crepuscular time periods. This pattern continued as such until the end of the week. The following week we reversed the pattern starting with the afternoon to evening crepuscular period. We used an instantaneous scan sample method (Heise et al. 2019) using tripod spotting scopes and binoculars (10 × 42) on lookouts, beaches, piers, and in a truck depending on the site. Only birds that were within 200 m of the shore were used for behavioral observations due to limitations in scanning equipment.

Prior to arrival, we recorded sunrise and sunset before each day's observation scans. Upon arrival and before each scan, we recorded the date, time, wind speed (m/s) and direction and air temperature (°C) using a handheld anemometer. We randomly decided the direction of the first scan by coin flip and alternated direction for each subsequent scan. During a 1‐h period, we scanned six separate times every 10 min to assure independence of observations (Jones et al. 2014; Heise et al. 2019). During the scan observations, we recorded behavior verbally into a voice recording device and later transcribed scans to data sheets. Behaviors were broken into six categories: flying, diving, swimming, preening, loafing, and sleeping (Heise et al. 2019). We characterized flight behavior as movement through the scanning area through the air as well as landing and taking off. Diving behavior, averaging ~10–20 s in duration (Lovvorn 1994; Stephenson 1994), was observed by watching scaup dive under the water and arrive to the surface. The pauses between diving bouts (< 15 s) or when an individual was seen eating was considered in the diving behavior category (Austin 1987; Byrkjedal 1997). Additionally, we gave 1–2 s of viewing time after all the individuals on the surface were recorded before continuing with the scan to try to account for individuals that were submerged underwater during a dive event. We observed swimming as back and forth lateral movement, preening as feather cleaning, bathing, and itching, loafing as awake with no movement, and resting as no movement with head tucked. Since equivalent energic costly behaviors have been shown to have significantly similar energy modifiers (McPherson 2020), we combined sleeping and loafing behaviors into one rest category and swimming and diving behaviors into one feeding category. Therefore, we categorized behaviors into four categories: flying, feeding, preening, and rest, because they represented the full range of energetic costs (McPherson 2020). Other behaviors such as agnostic, alert, and mating behaviors were not recorded as to keep consistent with the behaviors that were observed using ACC data as described below.

### 2.3 Trapping and Transmitter Implants

We trapped scaup between December–March 2021–2022 and 2022–2023 off the shore of Eastern Neck Wildlife Refuge located on the Chester River in Rock Hall, Maryland (39.010300, −76.211808; Figure 1) using closed barrier traps (Haramis et al. 1982). Each scaup received a United States Geological Survey (USGS) size six stainless steel band with a unique band number and were sexed, aged, and weighed to make sure scaup met the minimum requirements for internal transmitters (3% of body weight). We measured tarsus (cm) and wing cord (cm) for each individual to confirm Lesser Scaup identification from Greater Scaup ( *Aythya marila* ) according to Pyle (2008). To reduce detrimental effects that traditional external transmitter attachments have on diving ducks (Olsen et al. 1992), we used 30‐g GPS/GSM transmitters (OrniTrak‐I30 3 G; Ornitela, UAB, Vilnius, Lithuania) that were surgically implanted 64 individuals including 37 males and 27 females, and 38 individuals that were at least second year or younger (SY) and 26 that were aged as after second year (ASY) following procedures outlined in Olsen et al. (1992). Implant surgeries occurred at the Patuxent Veterinary Hospital, USGS Eastern Ecological Science Center (formally known as Patuxent Wildlife Research Center) located in Laurel, MD, ~141 km away from the trapping site. The internal transmitters were surgically implanted into the right caudal air sac. The surgery itself typically took 20–25 min and < 60 min from first starting anesthesia to recovery (Olsen et al. 1992). We released the scaup at the trapping site after a 3–4‐h recovery period. We set the transmitters to record location (longitude and latitude) every 60 min and an accelerometer *x*‐*y*‐*z* directional degree reading every 10 min for 5 s bursts at 10 Hz.

Specifically, when using internal transmitters, Mulcahy and Esler (1999) recommend censoring data for up to 14 days post release to minimize surgery effects that are associated with abnormal movement and behavior. However, this recommendation was determined using low frequency transmitter data that were acquired once every 3–6 days (Mulcahy and Esler 1999). With the advancement of telemetry and GPS transmitter technology, location data can be collected at higher rates leading to fine‐scale movement data. This can be used to identify more precise estimates of movement rates that may show abnormal patterns associated with transmitter surgical procedures and when those patterns return to normal. Using the hourly GPS location data, we calculated the average distance traveled (km/day) for up to 20 days post release using the “amt v. 0.2.1.0” package in RStudio (Dechen Quinn et al. 2012; Signer et al. 2023). We used these distances for each individual and a time‐series change point analysis with the “changepoint v. 2.2.4” package in RStudio to determine censored periods that corresponded to crucial times post‐release when significant movements were made (Edelhoff et al. 2016; Killick et al. 2022). However, in accordance with Mulcahy and Esler (1999), we only included individuals that survived > 14 days post‐release and stayed within the Maryland portion of the Chesapeake Bay for any further analysis. This resulted in 29 individuals with 17 males and 12 females, and 14 SY and 15 ASY.

### 2.4 Experimentally Categorizing and Classifying Behaviors Using ACC Data

We videotaped two scaup (one male and one female) in concrete dive tanks (2.5 m deep) from the EESC's seabird colony to categorize ACC data into behaviors. The dive tank is equipped with three windows located approximately halfway between the top and bottom of the tank. We fitted each scaup with the same internal transmitters using the same surgical procedures as those captured from the Chesapeake Bay (Olsen et al. 1992). Transmitter accelerometer *x*‐*y*‐*z* directional degree readings were recorded every 5 s at 10 Hz for 5 s bursts resulting in almost constant data collection. Each scaup was marked with different colored leg bands to help with identification once in the dive tank. Approximately 2 weeks prior to video recording, we reinforced previous training for the scaup to dive in the tank by using mealworms that would sink to the bottom of the tank to entice feeding behavior (de Leeuw et al. 1998). We placed a video camera next to a window and aligned it so that the full water column of the dive tank was in focus. We placed a small clock that displayed the hour, minute, and second on the bottom left side of the window, so that it always remained in view of the camera. Each video recording event lasted approximately 4–6 h (de Leeuw et al. 1998). In total, we videotaped 60 h of footage that resulted in 48 h of usable footage, meaning that at least one scaup was in view of the camera and was able to be identified based on the colored leg band (University of Delaware IACUC permit: 1385‐2021‐0 and 1385‐2022‐A, USGS ACUC permit: 2021‐16P). We categorized the videoed behaviors into the same categories using the same guidelines as with the scanning observations: resting (including loafing and sleeping behaviors), preening, and feeding (including swimming and diving behaviors). We matched the timestamp and the ACC data from the transmitters to the timestamp and performed behavior from the video footage to create a list of verified behaviors with their coordinating ACC signatures. We only recorded behaviors that accounted for ≥ 3 s out of the total 5 s the behavior was performed (Yu and Klaassen 2021). Since flight, which is the most energetically costly behavior (Morton et al. 1989; Gill et al. 2001; Cramer et al. 2012), could not be measured in the dive tank, we verified this behavior ACC's signature separately using the ACC data of the hourly GPS coordinates during migration events characterized by cross‐continental movement over land.

**FIGURE 2:**
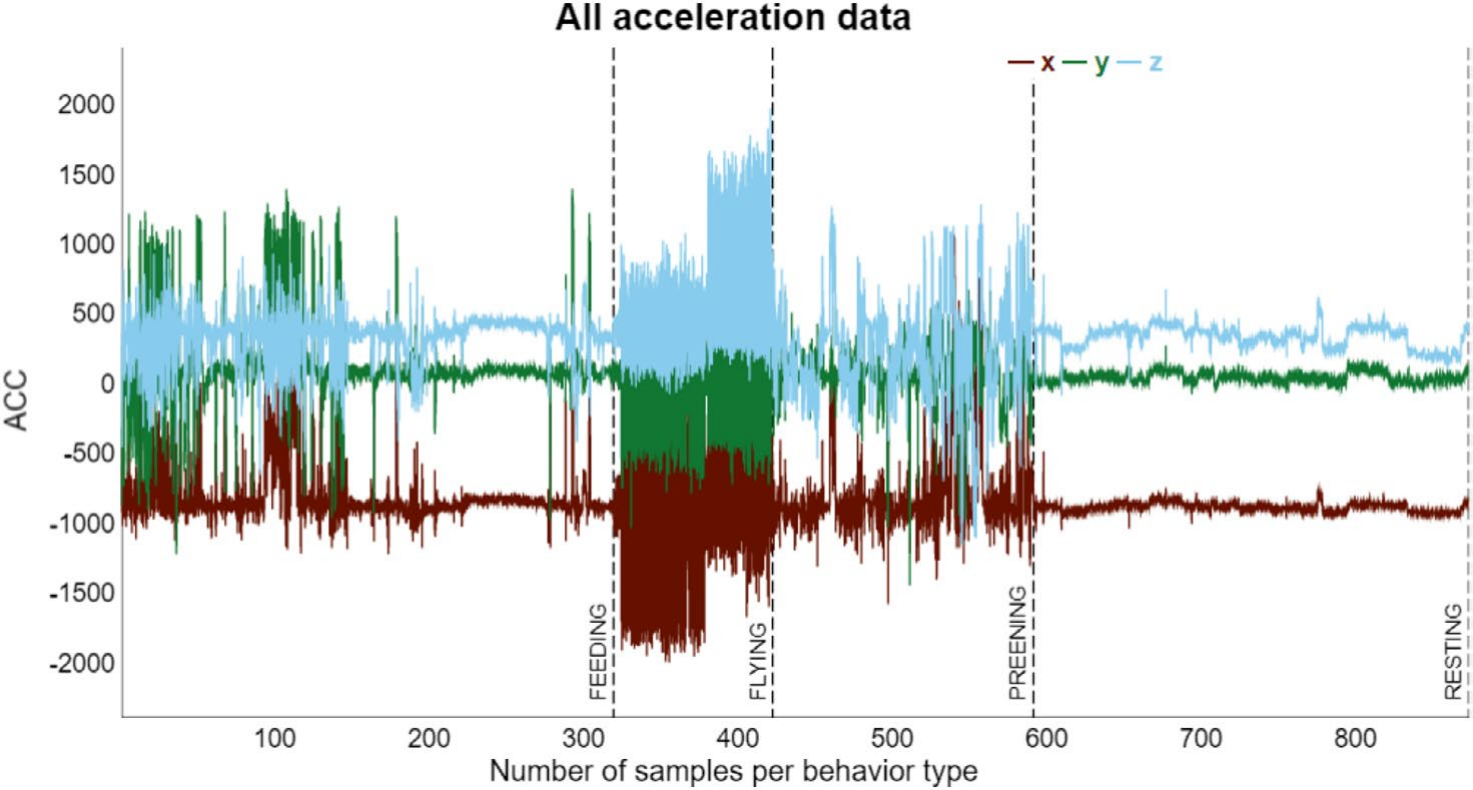
Visualization of video‐taped tri‐axial accelerometer data collected from a dive tank study performed at the US Geological Survey, Eastern Ecological Science Center, Laurel, Maryland, USA, of two captive bred Lesser Scaup (1 male and 1 female, *Aythya affinis* ) pooled and sorted by behavior type: Feeding (*n* = 319), flying (*n* = 103), preening (*n* = 169), and resting (*n* = 282), where flying behavior was verified using GPS location data from cross‐continental movement during migration periods during December–March 2021–2022 and 2022–2023 wintering periods.

Past research has documented that shorter burst duration (1–3 s) has minimized the chance of recording ACC data of mixed behaviors (Brown et al. 2013; Brandes et al. 2021). Following this, we modified all ACC data to account for the middle 3 s bursts at 10 Hz in an attempt to isolate pure behaviors. The ACC data were verified to include four behavior groups: feeding, resting, preening, and flying (Figure 2). To translate the continuous ACC data to behaviors, we used the “rabc v. 0.1.0” package that uses XGBoost distributed gradient‐boosted decision tree machine learning to analyze the relationships between the *x*‐*y*‐*z* coordinates using a total of 92 preprogrammed and added derived features and predictive models that were filtered using a 0.9 correlation coefficient threshold to prevent autocorrelation (Minerva Center for Movement Ecology 2014; Rast 2019; Yu 2021; Yu and Klaassen 2021). We used the ACC GPS derived flight data and the dive tank video data as training data that consisted of four behavior categories: flight (*n* = 103), resting (*n* = 282), preening (*n* = 169), and feeding (*n* = 319; swimming = 139 and diving = 180; Figure 2). The resulting top three features were used in the final predictive model to assign the behavior categories to the unclassified ACC *x*‐*y*‐*z* coordinates. All involved field and captive data collection and procedures were approved by the federally mandated University of Delaware Institutional Animal Care and Use Committee (permit: IACUC 1385‐2021‐0 and 1385‐2022‐A), the US Geological Survey—Eastern Ecological Science Center Animal Care and Use Committee (permit: ACUC 2021‐16P), and the US Geological Survey—Bird Banding Laboratory (permit: 06570).

### 2.5 DEE Calculations

We used scanning data and ACC data to calculate DEE using the following equation:

(1) $${\mathrm{DEE}}_{\mathrm{TE}24}=\sum_{\mathrm{hr}=1}^{n}\sum_{i=1}^{n}\left\{\left[\left(\mathrm{RMR}\times a_{i}\right)+C_{T}\right]\times t_{i}\right\}$$

where RMR represents the resting metabolic rate (15.89 kJ/bird/h; McPherson 2020),*a*_*i*_ represents the activity‐specific multiplier of RMR for a given behavior, *C*_T_ represents the cost of thermoregulation (kJ/h), and *t*_*i*_ represents the proportion of time spent in a specific behavior, informed by behavioral proportions obtained via instantaneous scan samples and accelerometry data.

We used the activity‐specific modifiers presented by McPherson (2020) where *a*_preening_ = 1.66, *a*_feeding_ = 1.85 (averaged between *a*_diving_ = 1.89 and *a*_swimming_ = 1.81) and *a*_resting_ = 1.05 (averaged between *a*_loafing_ = 1.10 and *a*_sleeping_ = 1.0). The activity‐specific multiplier for flight (*a*_flight_) was calculated using the following equation presented by Butler and Bishop (2000):

(2) $$P_{\text{flight}}=52.6\times M^{0.74}$$

where *P*_flight_ is the total flight power (*W*) and is calculated using the mass (kg) and converted to kJ/h and divided by the RMR to get the flight multiplier (*a*_flight_ = 9.2; Ladin et al. 2011).

The *C*_T_ is the cost of energy that is used for thermoregulation when the temperature is below a lower critical temperature (LCT). When the LCT was lower than 14.4°C (McEwan and Koelink 1973), we calculated the thermoregulatory energy expenditure using the equation derived from captive Lesser Scaup (McEwan and Koelink 1973).

(3) $$C_{T}=0.1392\left(T\right)$$

where *T* is the temperature subtracted from the LCT. If the temperature was above 14.4°C, we assumed that there were no additional thermoregulatory costs. We converted the oxygen consumption using the 20.1 kJ/h to 1 L O_2_/h conversion factor presented by Carey (1996). We calculated the behavior proportions for each scan (*t*_*i*_) and used Equation (1) to estimate hourly energy expenditure (HEE) for each observation. We multiplied the average HEE by the number of daylight hours (beginning of sunrise—end of sunset) to determine the DEE for the diurnal period. Additionally, assuming diurnal behavior was indicative of the full 24‐h period, we multiplied the average HEE by 24 to determine a single winter DEE value for scaup.

For the ACC data, we assigned one of the four behaviors to the unclassified ACC data for days that had ≥ 95% of all total bursts recorded (*n* = 191,186) using the results of the XGBoost decision tree analysis. Once the unclassified ACC coordinates were assigned one of the behaviors, we calculated the behavior proportions for each individual throughout the December–March of 2021–2022 and 2022–2023 wintering period. We paired the behaviors to the closest hourly ambient temperature (°C) and wind speed (m/s) collected from the closest weather station provided by the National Centers for Environmental Information, National Oceanic and Air Administration (NOAA) National Climatic Data Center (National Climatic Data Center 2023). We used the above equations to calculate HEE for each hour and summed to find DEE for full 24‐h estimates and averaged across all DEE estimates to calculate a single average DEE for the wintering periods of December–March of 2021–2022 and 2022–2023. We also calculated the DEE estimate for daylight hours by multiplying the average HEE by the average number of daylight hours, calculated using the “suncalc v. 0.5.1” RStudio package (Thieurmel and Elmarhraoui 2022), for each day in the sampling period as well as an estimate for the nocturnal sampling period.

### 2.6 Time‐Activity Budget and DEE Comparison Analysis

We first calculated the behavior proportions collected from all individual scaup observations that occurred during the 10‐min instantaneous scan samples (*n*_observations_ = 42,713; *n*_scans_ = 396) and those collected from the ACC data from 29 individuals (*n*_bursts_ = 191,186). We tested specifically the differences between scaup feeding and flying behaviors as these two behaviors have the greatest effect on DEE estimates because of their high energetic demands. As behaviors are generally nonparametric, we conducted Wilcoxon rank sum tests using the “rstatix v. 0.7.2” package using the ACC data to compare the two behavior groups between the diurnal and nocturnal time periods (Kassambara 2023). With the data having a truncated distribution toward 0, the traditional approach of using the rank‐based Wilcoxon rank sum test may also result in a lower power analysis (Wang et al. 2023). Therefore, we additionally performed binomial Generalized Linear Mixed Models (GLMM) with each scaup as a random effect in the “lme4 v. 1.1‐34” package for feeding and flying behaviors which allowed us to compare behavior groups to the different time periods for the 24‐h ACC data (Bates et al. 2023). Similarly, we performed a binomial GLM to compare the behavior proportions of feeding and flight between the instantaneous scan sampling and ACC transmitter methods. We used a two‐tailed hypothesis t‐test to examine if there is a significant difference between the 24‐h DEE estimates of the scanning methodology and the ACC methodology. We also used a two‐tailed hypothesis test to determine if the diurnal energy expenditure calculated from the scanning samples were different than those calculated from the ACC data. To compare daily energy expenditure to nocturnal energy expenditure, we used a two‐tailed hypothesis test to determine any significant differences between the two estimates. All statistical analysis was performed using RStudio v4.3.1 (R Core Team 2023).

## 3 Results

### 3.1 Training ACC Sampled Data

**FIGURE 3:**
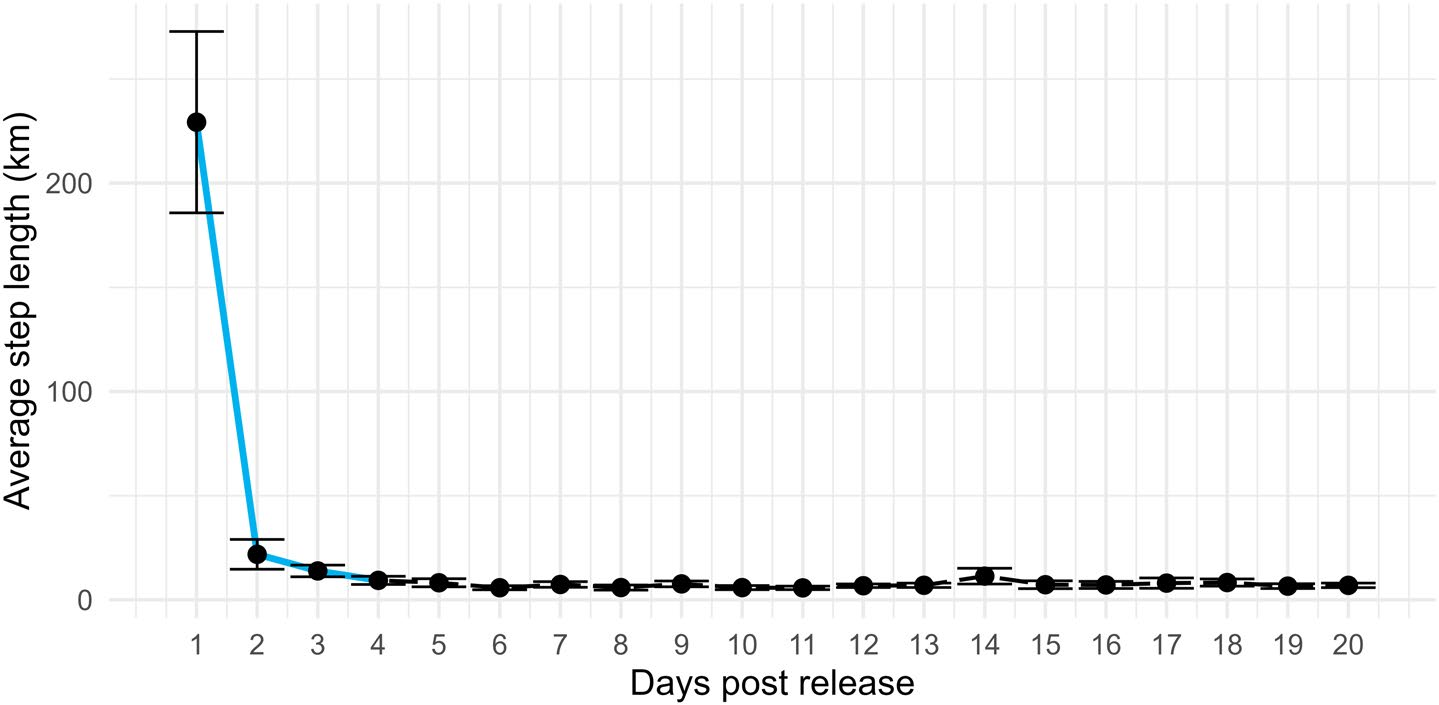
Line graph showing the average distance traveled (km) with SE bars for every individual Lesser Scaup (*Aythya affinis, n* = 29) that survived > 20 days post release with blue highlighted lines indicating that between days 1–2 and days 3–4 had the most significant displacement in movement for winters of 2021–2022 and 2022–2023, Chesapeake Bay, Maryland USA.

To train the ACC data, we wanted to remove potentially biased initial behaviors associated with transmitter implantation. The average total distance traveled for all 20 days was 390.1 ± SE 35.7 km. The greatest bout of movement occurred between days 1–2 where the average distance traveled per day went from 229.3 ± SE 36.2 km to 21.8 ± SE 6.0 km with an effect size of 0.9 (Figure 3). The second largest change in mean average distance traveled per day was between days 3–4 and changed from 13.9 ± SE 2.3 km to 9.3 ± SE 1.6 km/day with an effect size of 0.6 (Figure 3). The average distance traveled for the remaining 16 days was 7.2 ± SE 0.3 km. Therefore, all transmitter bursts were censored to exclude the first 4 days.

**FIGURE 4:**
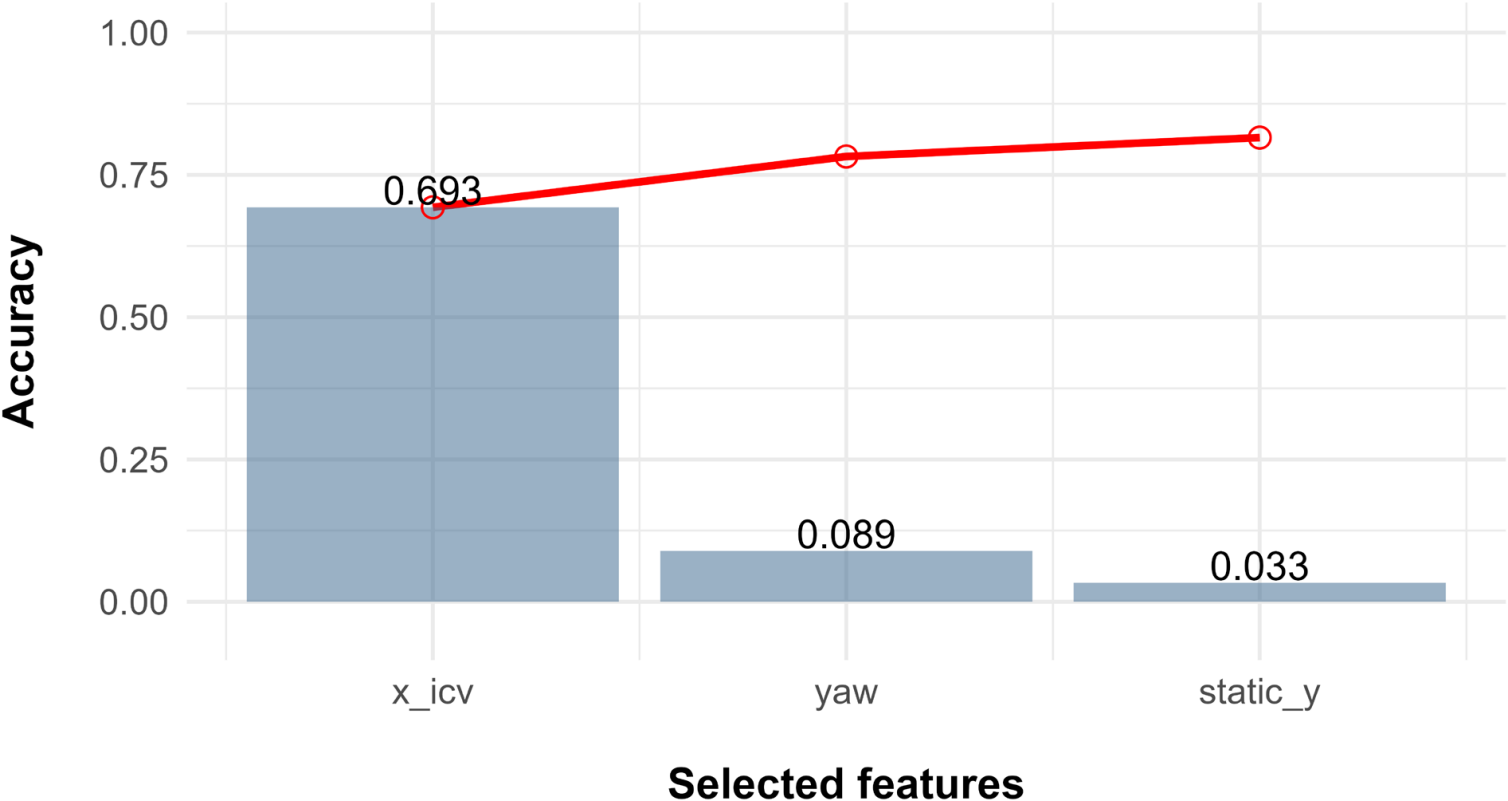
Top three features used in the XGBoost decision tree model for predicting four behaviors of tagged Lesser Scaup ( *Aythya affinis* ) using ACC transmitter data in the Chesapeake Bay, Maryland USA, 2021–2023, and the cumulative model accuracy of each predictor variable to the overall model (red line) where *x*_icv is the inverse co‐variance of the *x* coordinate, yaw the rotation around the z coordinate, and static_*y*, the absolute value of the running mean of the *y* coordinate.

**FIGURE 5:**
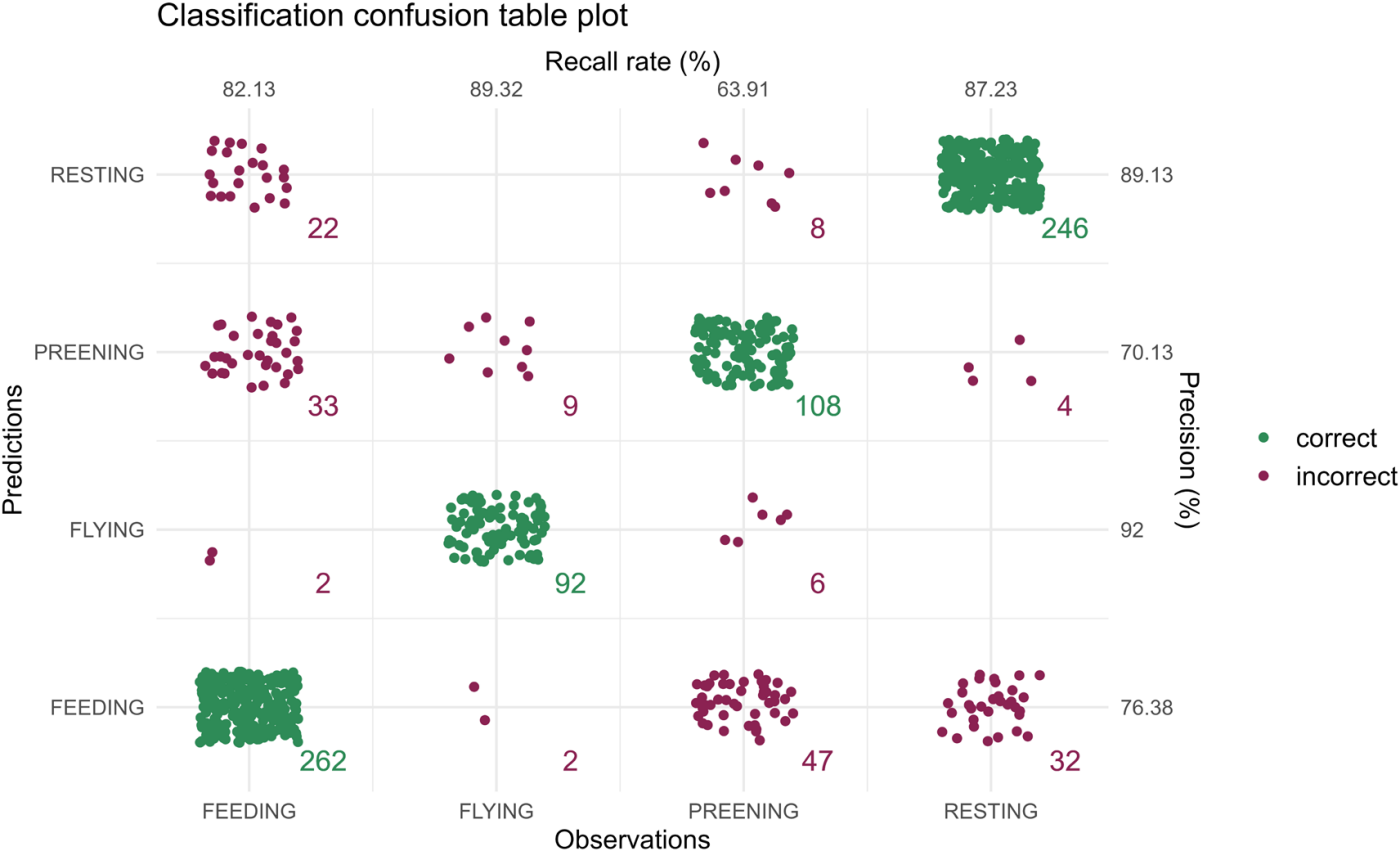
Confusion matrix detailing accuracy of each Lesser Scaup ( *Aythya affinis* ) behavior to be correctly identified given the XGBoost model with the top accurately identified behavior as flying, followed by resting, feeding, and lastly, preening, during the winters of 2021–2022 and 2022–2023, Chesapeake Bay, Maryland USA.

The top three data features for classifying behaviors using the gradient boosted machine model were *x*_icv, the inverse co‐variance of the *x* coordinate, yaw, the rotation around the *z* coordinate, and static_*y*, the absolute value of the running mean of the *y* coordinate (Figure 4). With these three summary statistics, the overall classification accuracy of the model was 81.5% (Figure 4). The F1 score is a performance measure that evaluates the accuracy of a model using the precision and recall scores for each variable (Zhang et al. 2015). Using the precision and recall values from the model confusion matrix (Figure 5), flying behavior had the highest F1 score (0.91) indicating that flight behavior was the most correctly identified. Resting behavior was the second most correctly identified behavior class (F1 = 0.88) followed by feeding behavior (diving and swimming, F1 = 0.79) and lastly, preening (F1 = 0.67).

### 3.2 Estimated Behaviors

Resting behavior (sleeping and loafing) had the highest proportion of time spent of all behaviors (69.6%) that were collected using the instantaneous scan sampling method (*n* = 42,713) followed by feeding (diving and swimming) (24.5%), flying (4.1%), and lastly preening (1.9%) (Table 1). The behavior proportions from the 24‐h ACC data (*n*_diurnal_ = 86,781; *n*_nocturnal_ = 104,405) from most to least occurring were preening (65.0%), feeding (diving and swimming) (29.1%), flying (3.5%), and resting (2.5%) (Table 1). During the diurnal hours, preening was the most occurring behavior (57.6%), followed by feeding (37.7%), flying (3.0%), and resting (1.7%) (Table 1). Preening was the highest representing behavior for the nocturnal hours (71.1%) followed by feeding (21.9%), flying (3.9%), and resting (3.1%) (Table 1).

**TABLE 1:** Contingency table for Lesser Scaup ( *Aythya affinis* ) outlining the percentages and total counts of all four behaviors between the ACC December–March 2021–2022 and 2022–2023 wintering periods, Chesapeake Bay, Maryland USA, for both diurnal and nocturnal time portions of the day and the total for all diurnal counts with SE for scanning observations.

	Feeding	Flying	Preening	Resting
**ACC**				
Diurnal	37.7% (32,715)	3.0% (2607)	57.6% (50,003)	1.7% (1456)
Nocturnal	21.9% (22,838)	3.9% (4063)	71.1% (74,232)	3.1% (3272)
Total	29.1% (55,553)	3.5% (6670)	65.0% (124,235)	2.5% (4728)
**Scanning observations**				
Diurnal	24.5% ± 13.3% (10,149)	4.1% ± 2.2% (1676)	1.9% ± 0.9% (867)	69.6% ± 45.2% (30,021)

Our results from the Wilcoxon ranks sum test comparing the behavior proportions between the diurnal and nocturnal time periods indicated that both feeding (*p* < 0.001) and flying (*p* < 0.001) were significantly different with small effect sizes (*r*_feeding_ = 0.1; *r*_flying_ = 0.03). Similar results were shown from our test comparing the diurnal and nocturnal behaviors derived from the ACC data where feeding behavior was significantly different between the diurnal and nocturnal time periods and showed that more feeding occurred during the daylight hours (37.7%) than in the nocturnal periods (21.9%, *p* < 0.001). Additionally, the binomial model for flying behavior showed that there was also a significant difference between flight behavior in the two time periods with more flight behavior during the nocturnal hours (3.9%) than in the diurnal time period (3.0%, *p* < 0.001). Our results from the binomial GLM comparing the two methodologies for the diurnal feeding behavior indicated that there was a significant difference (*p* < 0.001) and that the ACC estimated behaviors (37.7%) were observed more often than for the scanning methods (29.1%). The diurnal flight behavior also had significant differences (*p* < 0.001) between the two methods with scanning observations (3.5%) being more often recorded than with the ACC transmitter data (3.0%).

### 3.3 Estimating Energy Expenditure

The average hourly energy expenditure calculated from instantaneous scan samples for daylight hours (*n* = 42,713) was 53.5 ± SE 1.3 kJ/bird/h (95% CI: 51.0–56.1 kJ/bird/h). The extrapolated DEE for a 24‐h period (assuming the diurnal behavior was representative of the 24‐h period) was 1285.1 ± SE 30.9 kJ/bird/day (95% CI: 1224.5–1345.7 kJ/bird/day). The average ACC diurnal HEE (*n*_diurnal_ = 86,781) was 65.0 ± SE 2.2 kJ/bird/h (95% CI: 60.6–69.3 kJ/bird/h). The average ACC nocturnal HEE (*n*_nocturnal_ = 104,405) was 107.8 ± SE 11.0 kJ/bird/h (95% CI: 86.1–129.5 kJ/bird/h). The average HEE across the full 24‐h period was 62.3 ± SE 0.3 kJ/bird/h (95% CI: 61.7–62.9 kJ/bird/h). Nocturnal energy estimates were significantly greater than diurnal energy estimates (*t* = −65.5, *p* < 0.001). The DEE for the full 24‐h period was 1496.0 ± SE 7.4 kJ/bird/day (95% CI: 1481.4–1510.6 kJ/bird/day).

Our results of the two‐tailed hypothesis *t*‐test comparing the diurnal hourly energy expenditure estimates between the two methods indicated that there was a significant difference (*t* = −9.6, *p* < 0.001), with estimates being greater from the ACC data (65.0 ± SE 2.2) than those estimated through scanning observations (53.5 ± SE 1.3, *p* < 0.001). Comparing the full 24‐h DEE estimate between the scanning and ACC methods, our results showed that there was a significant difference (*t* = −6.6, *p* < 0.001) with the DEE estimates from the ACC data (1496.0 ± SE 7.4) yielding a greater estimate than those from the scanning observations (1285.1 ± SE 30.9).

## 4 Discussion

Incorporating advanced technology, such as telemetry devices, into wildlife research has provided several valuable insights into animal behavior and movement. Scaup are a species that can greatly benefit from these innovations, which allow for continuous data collection during visually difficult time periods and from elusive locations. The aim of this research was to provide new methods to calculate DEE and assess wintering Lesser Scaup behavior. By using the ACC data to maximize behavior observations, a full 24‐h daily energy budget was estimated, and we found that nocturnal energy expenditure was significantly greater than diurnal energy expenditure estimates and that the ACC transmitter data estimated a significantly greater DEE and HEE than instantaneous scan sampling. Additionally, there were significant differences between behaviors during the diurnal and nocturnal time periods which greatly influenced their calculated diurnal and nocturnal HEE and added evidence to support the need for nocturnal behavior observations. The bias correction of an additional 211 kJ needed for a bird/day translates into an extra day of energy needed per week.

When performing behavior‐based research and analysis, the assumption that diurnal proportions of the day are representative of the nocturnal behaviors should be met with skepticism. The ACC observations yielded a significantly higher DEE estimate for the 24‐h period than the estimate calculated from the scanning samples. The diurnal scan samples occur when temperatures are typically warmer than nocturnal temperatures which impacts the energetic adjustments for LCT. Thus, when the scan sample HEE was being extrapolated to a 24‐h estimate, nocturnal thermoregulation was not being accounted for. This was shown in our study, where the nocturnal ACC DEE estimates were significantly greater than the diurnal estimates. Findings presented by Jorde and Owen Jr. (1988), state that some waterfowl species perform different behaviors over the nocturnal period. Specifically, warmer temperatures may allow waterfowl to spend more time performing different behaviors than when the temperatures reach below the LCT threshold (Jorde et al. 1984). Furthermore, these daily temperature changes can heavily influence the amount of thermoregulation occurring and increase the amount of energy needed for a single day. For instance, for ring‐neck ducks ( *Aythya collaris* ) wintering in Florida, USA, where the mean diurnal and nocturnal ambient air temperatures are higher than those around the Chesapeake Bay, the *DEE* estimate for the 24‐h period was considerably lower than the ACC and scanning estimates (Jeske and Percival 1995). Incorporating other biological and microclimate effects, such as body morphometrics and wind velocities, may also provide more robust estimates for energy expenditure (McKinney and McWilliams 2005; Livolsi et al. 2015).

For the marked scaup in this study, both the results from the tests comparing the feeding and flying proportions between diurnal and nocturnal time periods indicate that feeding occurred significantly more during the daylight hours and there was more flight behavior during the night. As previously mentioned, these differences between the two time periods have great impact on these estimates because more energy is required to perform these behaviors during colder nocturnal temperatures. As scaup are a waterfowl species that can be difficult to see during the night, using other methods to collect behavioral observations other than traditional scanning procedures has proven to be beneficial to capture high energy behaviors.

Our findings documented feeding behaviors as the second most prevalent behavior in the diurnal period in both the scanning and ACC observational methods, and our estimates fell within range of previous studies (~20%–48%) estimating feeding behavior (Christopher and Hill 1988; Bergan et al. 1989; Adair et al. 1996; Custer et al. 1996; Poulton et al. 2002; Herring and Collazo 2005). Although our results showed that feeding performed more often during diurnal hours, past literature suggests that scaup feed more often nocturnally due to outside influences that were not accounted for during our study. For instance, Nilsson (1970) found scaup feeding was impacted by temperature and potential disturbances on the foraging grounds. Bergan et al. (1989) and Custer et al. (1996) documented that specific behaviors such as foraging and locomotion can change throughout the wintering season and may be impacted by human disturbances and environmental stimuli such as food resources, inter‐ and intraspecific competition, weather, lunar and tidal cycles, as well as noise pollution and light pollution (Korschgen and Dahlgren 1992; Cabrera‐Cruz et al. 2018). Coupling the 24‐h observational monitoring from the ACC data with these additional outside influences may address these confounding results and share insight on feeding behaviors of scaup, especially during periods and at locations where visibility during nocturnal hours is low or non‐existent.

Contrary results from the scanning and ACC behavioral time budgets indicate that preening was observed the least often with the scanning samples and was observed the most often with the diurnal ACC data. The opposite relationship was shown with resting behavior as it was observed the most using scan sampling and the least with the diurnal ACC data. Neither the scanning nor the diurnal ACC observations fell within previous research conducted that estimated preening (or noted as comfort) ranged between ~4% and 18% (Christopher and Hill 1988; Bergan et al. 1989; Adair et al. 1996; Poulton et al. 2002). Past literature suggests conflicting patterns regarding preening and resting behaviors of Lesser Scaup between diurnal and nocturnal hours (Bergan et al. 1989; Adair et al. 1996), which is similar to the findings presented in this paper. In the case of the opposing performance rates between the two methods, one of the possible explanations may result from collecting behavior observations from different ranges from shore. Behaviors that were observed using scan sampling were constricted to 200 m from shore whereas the ACC data did not have the same limitations and could be observed from any distance. Since scaup have been shown to rest in open water areas closer to shore (Bergan et al. 1989), the resting observations collected from the scan sampling may have been inflated since not all behaviors performed from all distances were represented in this sampling method. Another explanation for these opposing performance rates may be from difficulty to interpret and assign ACC behaviors that are confounded by external forces, such as waves (Halsey et al. 2011). For stationary behaviors or behaviors that exhibit a consistent velocity, such as resting and swimming, ACC readings may have difficulties deciphering out these behaviors (Williams et al. 2017). Considering that the trial data were taken from a controlled setting, the impact of a tidal environment on the *x*‐*y*‐*z* coordinates was not measured—potentially leading to the misclassification of behaviors. Past research suggests that scaup rest more often in open estuarine habitats (Herring and Collazo 2005). Thus, if a scaup was resting in an open water area, the impact of the waves may have shown a different ACC reading and labeled the behavior as something other than resting. Furthermore, for ACC data, functionally similar behaviors can be difficult to differentiate and can be mislabeled (Tatler et al. 2018). During the video behavior classification, any behavior that exhibited “comfort,” i.e., bathing, feather cleaning, and itching, were labeled as a broad “preening” category. If an ACC reading in the broad preening category had a similar *x*‐*y*‐z statistical summary relationship to those in the flying, feeding, or resting categories, that behavior may have been misclassified. This may have inflated the predictions associated with the preening behavior while simultaneously underestimating resting behavior. Brandes et al. (2021) found a similar phenomenon when trying to differentiate between specific behaviors in giraffes (*Giraffa cameloparadalis*) and noted that highly overlapping values bordering single axes per behavior category may cause possible confusions during classification.

As previously stated, the behavior modifiers and trial behavior observations used for the ACC data were derived from an environment set to stable conditions and did not incorporate external environmental variables such as water movement or waves. With the potential bias leading to the misclassification of behaviors, the DEE may be miscalculated and be under‐ or overestimating estimates. However, scaup residing in these alternating conditions may require additional energetic requirements that have been unaccounted for in past DEE estimates. Since it requires greater amounts of energy to move and remain at rest in dynamic environments, such as water currents and wave‐surge flows (Woakes and Butler 1983; Schakmann and Korsmeyer 2023), the estimate we present in this study that incorporated more energetically taxing behaviors, that is preening, may be a more realistic representation of the amount of energy expended daily for scaup wintering in the Chesapeake Bay because of the strenuous environmental conditions. In the case of scaup wintering in the Chesapeake Bay, it may be a better solution to use additional or different sensors that measure angular rotation such as gyroscopes or magnetometers (Martín López et al. 2016; Williams et al. 2017).

When comparing the two methods, it should be noted that both have advantages and disadvantages associated with both. For diurnal scanning, we observed behaviors from many individuals without needing to perform intrusive handling and surgery procedures and without the added monetary costs of transmitters. However, these data were only able to be collected in locations that allowed public access and when individuals were within our 200 m equipment threshold in which visibility was not obstructed by precipitation or choppy waves. Using the ACC data provided by the internal transmitters gave us access to behaviors that were performed during all weather and environmental conditions, in all locations in the Chesapeake Bay, and for full 24‐h cycles. While the initial field work of trapping and the transmitter implants required much effort between multiple collaborators, the data collection post‐surgery was consistent and provided us with extensive behavior observations that we would not have been able to observe otherwise. Yet, we were only able to acquire behaviors from 29 individuals, which is a small sample and may not be representative of the population, required additional field work to obtain ground‐truthed behaviors to run our analysis, and had higher expenses from the transmitter and trapping equipment. Regardless of the method used, it is essential to understand the biases that come with each form of data collection and account for these when estimating behaviors and energy expenditure.

Along with the technological advancements of data collection for wildlife comes the ability for wildlife researchers to gain meaningful knowledge and add to our current understandings of basic wildlife ecology. In this study, we presented only a few ideas of ways that radiotechnology can improve our data collection and new ways to use these data to re‐evaluate prevailing questions. Using ACC data hold powerful capabilities that allow researchers to rely less on extrapolated energy expenditure based on visually accessible behaviors and instead focus our efforts on calculating determinate 24‐h energy expenditure. For scaup in the Chesapeake Bay, 24‐h data behavior observations were successfully collected using the ACC tri‐axial data from GPS/GSM transmitters and showed that behaviors performed during the diurnal hours were not significantly similar to those during nocturnal hours. With this knowledge, we were able to calculate a full 24‐h energy expenditure estimate for wintering scaup that was significantly greater than the diurnal extrapolated estimate calculated using scanning observations showing just one example of how this technology can refine wildlife data collection.

## Author Contributions

**Hannah L. Schley:** data curation (lead), formal analysis (lead), investigation (lead), methodology (equal), writing – original draft (lead), writing – review and editing (equal). **Christopher K. Williams:** conceptualization (equal), data curation (supporting), formal analysis (supporting), funding acquisition (lead), investigation (supporting), methodology (equal), project administration (lead), resources (lead), supervision (lead), writing – original draft (supporting), writing – review and editing (equal). **Josh Homyack:** conceptualization (supporting), methodology (supporting), writing – review and editing (supporting). **Bill Harvey:** conceptualization (supporting), methodology (supporting), resources (supporting). **Glenn H. Olsen:** methodology (supporting), writing – review and editing (supporting). **Sharon Johnson:** methodology (supporting), writing – review and editing (supporting).

## Funding

This work was supported by Maryland Department of Natural Resources and Ducks Unlimited. Additional funding was provided by the US Department of Agriculture Hatch (DEL00774) and the University of Delaware Waterfowl and Upland Gamebird Center.

## Ethics Statement

All involved field and captive data collection and procedures were approved by the federally mandated University of Delaware Institutional Animal Care and Use Committee (permit: IACUC 1385‐2021‐0 and 1385‐2022‐A), the US Geological Survey—Eastern Ecological Science Center Animal Care and Use Committee (permit: ACUC 2021‐16P), and the US Geological Survey—Bird Banding Laboratory (permit: 06570).

## Conflicts of Interest

The authors declare no conflicts of interest.

## Data Availability

Our data are available at Dryad and the link is: https://doi.org/10.5061/dryad.tx95x6b8v.
